# Transitions of Care Coordination Intervention Identifies Barriers to Discharge in Hospitalized Stroke Patients

**DOI:** 10.3389/fneur.2021.573294

**Published:** 2021-05-17

**Authors:** William Denney Zimmerman, Rachel E. Grenier, Sydney V. Palka, Kelsey J. Monacci, Amanda K. Lantzy, Jacqueline A. Leutbecker, Xue Geng, Mary Carter Denny

**Affiliations:** ^1^Department of Neurology, MedStar Georgetown University Hospital, Washington, DC, United States; ^2^Massachusetts General Hospital, Interventional Radiology, Boston, MA, United States; ^3^Department of Neurology, University of Pittsburgh Medical Center, Pittsburgh, PA, United States; ^4^Department of Biostatistics, Bioinformatics and Biomathematics at Georgetown University Medical Center and Lombardi Comprehensive Cancer Center, Washington, DC, United States

**Keywords:** stroke, length of stay, nurse navigator, transitions of care, patient satisfaction

## Abstract

**Background:** Prolonged hospital lengths of stay increase costs, delay rehabilitation, and expose acute ischemic stroke patients to hospital-acquired infections. We designed and implemented a nurse-driven transitions of care coordinator (TOCC) program to facilitate the transition of care from the acute care hospital setting to rehabilitation centers and home.

**Methods:** This was a single-blinded, prospective, randomized pilot study of 40 participants to evaluate the feasibility of implementing a TOCC program led by a stroke nurse navigator in hospitalized acute ischemic stroke patients. The intervention consisted of a stroke nurse navigator completing eight specific tasks, including meeting with stroke patients and their families, facilitating communication between team members at multi-disciplinary rounds, assisting with referrals to rehabilitation facilities, providing stroke education, and arranging stroke clinic follow-up appointments, which were confirmed to be completed by independent study personnel. The primary outcome was to assess the feasibility of the program. The secondary outcomes included comparing hospital length of stay (LOS) and patient satisfaction between the TOCC and usual care groups. We also explored the association between patient-level variables and LOS.

**Results:** The TOCC program was feasible with all pre-specified components completed in 84.2% (95% CI: 60.4–96.6%) and was not significantly different from the assumed completion rate of 75% (*p* = 0.438). There was no significant difference in median LOS between the two groups [TOCC 5.95 days (4.02, 9.57) vs. usual care 4.01 days (2.00, 10.45), false discovery rate (FDR)-adjusted *p* = 0.138]. There was a trend toward higher patient median satisfaction in the TOCC group [TOCC 35.00 (33.00, 35.00) vs. usual care 30 (26.00, 35.00), FDR-adjusted *p* = 0.1] as assessed by a questionnaire at 30 days after discharge. The TOCC study allowed us to identify patient variables (gender, insurance, stroke severity, and discharge disposition) that were significantly associated with longer hospital LOS.

**Conclusion:** A TOCC program is feasible and can serve as a guide for future allocation of resources to facilitate transitions of care and avoid prolonged hospital stays.

## Introduction

Each year, 795,000 strokes occur in the United States. Although the mortality from stroke has steadily declined over the past 10 years, the incidence of stroke continues to rise, which is driven primarily by an aging population (1). Stroke remains the leading cause of severe adult disability, with 75% of stroke survivors having limb weakness, 30% having language impairment, and up to 65% having cognitive impairment (2–4). Approximately two-thirds of individuals who suffer a stroke will survive and require rehabilitation after discharge from the acute care hospital setting (5). The total annual cost of stroke in the United States, including direct medical costs and indirect lost productivity, is currently estimated to be $120 billion and is projected to double to $240.7 billion by 2030 (6).

The average hospital length of stay for patients discharged with the principal diagnosis of stroke is 4.7 days (7). Prolonged hospital stays in stroke survivors cause delays in initiating rehabilitation and can increase the overall costs, with the average direct cost of inpatient hospital stays for stroke patients reaching 13.8 billion in 2013–2014, with a steady increase over the previous 15 years (7). Earlier initiation of high-level rehabilitation therapy is associated with improved functional outcomes after stroke (8). Moreover, stroke survivors who have new physical and cognitive impairments develop limitations in their ability to schedule follow-up appointments for post-stroke care (9). The National Quality Forum and Institute of Medicine have identified transitions of care from acute care hospitals to other care facilities and home as national priority (10–12). Interventions are needed to avoid unnecessary delays in acute care stroke hospitalization and facilitate the transition of care to rehabilitation facilities and home.

In this study, a nurse-led transitions of care coordination (TOCC) program was developed to facilitate the completion of acute care stroke evaluations, referrals to rehabilitation facilities, and stroke clinic follow-up. In this pilot study, we aimed to (1) evaluate the feasibility of implementing a TOCC program in patients admitted for primary diagnosis of acute ischemic stroke (AIS), (2) assess whether the TOCC program was associated with any difference in length of stay (LOS) or patient satisfaction, and (3) explore patient-level variables associated with prolonged hospital length of stay.

## Methods

### Study Design and Setting

We conducted a prospective, randomized pilot study to evaluate the feasibility of implementing a TOCC program led by a stroke nurse navigator in patients hospitalized with AIS. All patients were admitted to the stroke service at our institution, which is an academic, comprehensive stroke center (CSC) located in an urban center. The study participants were enrolled from April 2018 to February 2019. The study protocol was reviewed and approved by the institutional review board with IRB #2017-0621. The study is registered on Clinicaltrials.gov with clinical trial ID NCT04434638.

### Participants

Patients ≥ 18 years and admitted to the stroke service with a primary diagnosis of AIS were eligible. The diagnosis of AIS was confirmed radiographically on brain imaging and on clinical evaluation by a stroke neurologist. Patients with a primary diagnosis of subarachnoid hemorrhage, intracerebral hemorrhage, transient ischemic attack, or stroke mimic were excluded. Patients admitted under observation status were also excluded.

### Intervention

We developed the TOCC program to aid in the completion of the diagnostic evaluations as well as in the transition out of the acute care hospital setting. Multiple stakeholders, including stroke attending physicians, neurology trainees, nurses, case managers, physical and occupational therapists, and speech language pathologists, met to determine specific barriers to discharge for each individualized service offered. Based on these barriers noted by each level of provider, we created a detailed structure of what tasks must be carried out and how these could be easily facilitated. At our facility, because our stroke nurse navigator was already trained in acute evaluation and management of AIS as well as the nuances of stroke work-up, we opted to utilize her skill set in the TOCC program. In the TOCC intervention, the stroke nurse navigator completed eight specific tasks: (1) met the patient and family within 48 h of admission, (2) identified patient home location and insurance status, (3) coordinated communication between treating providers (neurologists, cardiologists, etc.) regarding pending diagnostic tests, (4) followed up physical, occupational, and speech therapy teams' recommendations for rehabilitation, (5) attended daily multi-disciplinary rounds, (6) facilitated referrals to acute and subacute rehabilitation facilities with case managers, (7) assisted bedside nurses in providing tailored stroke education and discharge instructions to patients and families, and (8) arranged stroke clinic follow-up appointments. The completion of these tasks were confirmed by both the nurse navigator and an independent study team member and were tracked using REDCap.

### Allocation and Blinding

Upon admission, the patients were randomly assigned in a 1:1 ratio to either the TOCC group (intervention) or the usual care group (control). Randomization was completed using a random number generator in the REDCap randomization module to reduce selection bias and equalize independent variables across the two groups. The patients were blinded to their group assignment. The stroke nurse navigator and stroke physicians involved in the care of patients were not blinded to the assignment group. The stroke physicians and study investigators were blinded to the outcome measures of feasibility, length of stay, and patient satisfaction.

### TOCC and Usual Care Groups

Patients in the TOCC group had their care coordinated by a stroke nurse navigator including the eight tasks specified above. Patients in the usual care group, which served as the control, received the current, ongoing method of care coordination by members of the multi-disciplinary stroke team. In the usual care group, there is no central point of contact for the eight care coordination tasks detailed above. The physicians, nurses, rehabilitation therapists, and case managers are individually responsible for talking to patients and their families/caregivers about the different aspects of the plan of care. The current practice is that members of this multi-disciplinary team meet with each other every weekday morning to discuss the discharge plan of care for each stroke patient on the inpatient stroke service.

### Outcomes

The primary outcome was feasibility of implementing a TOCC program, which was defined as completion of all eight TOCC program tasks by the stroke nurse navigator in at least 75% of the intervention group patients. The time in minutes spent on the TOCC intervention by the nurse navigator was also measured prospectively.

The secondary outcomes were LOS as measured by number of days in the hospital and patient satisfaction at 30 days after discharge in the TOCC group vs. usual care group. Patient satisfaction was determined using a questionnaire that assesses multiple facets of inpatient care and discharge logistics, including key variables such as overall care, secondary stroke prevention education, blood pressure management, and follow-up arrangements (Table 1). Scores in the individual categories ranged from 1 to 5, with 1 representative of very unsatisfied and 5 representative of very satisfied (maximum score, 35). The questionnaire was completed by phone with the patient by the stroke nurse navigator, who was unblinded to the group allocation, after discharge. If the patient was unable to complete the questionnaire, their primary caregiver was utilized as a surrogate. Our questionnaire is modeled after the Satisfaction With Stroke Care-19 assessment, which showed adequate reliability and validity in a subset of 166 stroke patients in the Netherlands in assessing satisfaction with hospital-based care and the transition 6 months after discharge (13). We wished to ensure the delivery of inpatient stroke care that supports patients and caregivers by meeting their needs and demands, with direct correlation with patient satisfaction and quality of care received and compliance to future management regimens (13).

**Table 1:** Patient satisfaction survey.

**Category**	**Stroke-specific patient feedback questions**
Rehabilitation	• Did OT, PT, or SLP discuss your rehabilitation plan with you? • On a scale of 1–5 (1 = very unsatisfied, 2 = unsatisfied, 3 = neutral, 4 = satisfied, 5 = very satisfied) how satisfied were you with the discharge rehabilitation plan?
Blood pressure	• Did you receive instructions related to blood pressure control and its importance to prevent future strokes? • On a scale of 1–5 (1 = very unsatisfied, 2 = unsatisfied, 3 = neutral, 4 = satisfied, 5 = very satisfied) how satisfied were you with the blood pressure instructions?
Care	• Did you feel that you received good or exceptional care following your admission for a stroke? • On a scale of 1–5 (1 = very unsatisfied, 2 = unsatisfied, 3 = neutral, 4 = satisfied, 5 = very satisfied) how satisfied were you with your overall care?
Discharge	• Did you have discharge instructions explained to you more than once? • On a scale of 1–5 (1 = very unsatisfied, 2 = unsatisfied, 3 = neutral, 4 = satisfied, 5 = very satisfied) how satisfied were you with the discharge instructions that were provided?
Diet and exercise	• Were you provided with teaching materials on diet and exercise? • On a scale of 1–5 (1 = very unsatisfied, 2 = unsatisfied, 3 = neutral, 4 = satisfied, 5 = very satisfied) how satisfied were you with the diet and exercise teaching materials?
Physician	• Did the stroke physician provide clear information about your plan of care? • On a scale of 1–5 (1 = very unsatisfied, 2 = unsatisfied, 3 = neutral, 4 = satisfied, 5 = very satisfied) how satisfied were you with the information received from the stroke physician?
Follow-up	• Did you have a follow-up appointment made or were you given instructions on how to schedule an appointment? • On a scale of 1–5 (1 = very unsatisfied, 2 = unsatisfied, 3 = neutral, 4 = satisfied, 5 = very satisfied) how satisfied were you with the process of obtaining a follow-up follow-up appointment?

*If no, then 0 points. If yes, then one to five points based on the level of satisfaction with each category*.

### Exploratory Analysis

For exploratory analysis, we also examined the association between patients' demographics and clinical variables and LOS within the entire cohort (both TOCC group and usual care group). Patient-level variables that were analyzed included age, gender, race, insurance status, Charlson comorbidity index (CCI), home distance from hospital, admission National Institutes of Health Stroke Scale (NIHSS), discharge modified Rankin Scale score (mRS), discharge disposition (home vs. home with home health vs. acute rehabilitation vs. subacute rehabilitation vs. deceased), time between admission and final echocardiogram result, speech language pathology (SLP) evaluation, physical therapy/occupational therapy (PT/OT) evaluation, and difference between date medically ready for discharge and actual discharge. Apart from baseline characteristics, most of these variables are key barriers that must be completed before discharge based on the American Heart Association/American Stroke Association guidelines (14).

### Sample Size

Given that this is a feasibility study, the primary objective is to assess the feasibility of this program to be implemented in an already complex acute care hospital setting. Based on the volume of AIS patients admitted to the stroke service at our academic medical center annually as well as the capacity of the stroke nurse navigator to complete the TOCC program tasks in addition to his or her day-to-day responsibilities, we estimated that 40 patients could be enrolled and followed up during a 12-month period (20 patients in each group). This sample size would allow us to assess feasibility and provide preliminary data to design a future large randomized controlled trial. This pilot study was not powered to detect a difference in LOS or patient satisfaction.

### Statistical Analysis

Descriptive analyses, including baseline characteristics (Table 2), were reported using frequencies and proportions for categorical variables and median and interquartile range (IQR) for continuous variables. An exact confidence interval of the completion rate of TOCC group based on a binomial distribution was obtained, and exact binomial test was used to compare the completion rate of TOCC group to the assumed completion rate of 75%. Fisher's exact test was used to compare the categorical secondary outcome of patient satisfaction between TOCC and Usual Care group. Wilcoxon rank-sum test was used for the continuous secondary outcome of hospital length of stay. The false discovery rate (FDR) method was used to control for multiple test problems for secondary outcomes.

**Table 2:** Baseline characteristics and descriptive analyses.

		**Usual care**	**TOCC**
Sample size (*n*)		21	19
TOCC Complete (%)	No	0 (NA)	3 (15.8)
	Yes	0 (NA)	16 (84.2)
Total time spent by TOCC (median in minutes, IQR)		NA (NA, NA)	105.00 (75.00, 127.50)
Length of stay (median in days, IQR)		4.01 (2.00, 10.45)	5.95 (4.02, 9.57)
Total satisfaction score (median, IQR)		30.00 (26.00, 35.00)	35.00 (33.00, 35.00)
Age on admission (median, IQR)		66.00 (58.00, 71.00)	74.00 (67.00, 84.50)
CCI score (median, IQR)		4.00 (3.00, 7.00)	6.00 (5.00, 7.00)
Gender (%)	Female	8 (38.1)	10 (52.6)
	Male	13 (61.9)	9 (47.4)
Race (%)	Black/African American	9 (42.85)	10 (52.6)
	Hispanic/Latino	0 (0.0)	1 (5.3)
	Not reported	3 (14.3)	2 (10.5)
	White/Caucasian	9 (42.85)	6 (31.6)
Premorbid mRS (%)	0	17 (80.9)	13 (68.4)
	1	3 (14.3)	3 (15.8)
	2	0 (0.0)	2 (10.5)
	3	1 (4.8)	1 (5.3)
NIHSS [median (IQR)]		6.00 (2.00, 13.00)	7.00 (2.50, 15.50)
Insurance type (%)	Commercial Insurance	6 (28.6)	3 (15.8)
	Medicaid	2 (9.5)	1 (5.3)
	Medicare	13 (61.9)	15 (78.9)

*TOCC, transitions of care coordinator; CCI, Charlson comorbidity index; mRS, modified Rankin scale; NIHSS, National Institutes of Health Stroke Scale*.

For exploratory analysis, we examined the association between patient-level variables, described above, and LOS within the entire cohort (both TOCC group and usual care group). Wilcoxon rank-sum test or Kruskal–Wallis test was used to determine the association between LOS and categorical patient-level variables, and Spearman's correlation coefficient was calculated to determine the association between LOS and continuous patient-level variables.

All tests are two-sided at a significant level of 0.05. All analyses were performed using statistical software RStudio (version 0.99.902).

## Results

### Feasibility

From April 2018 to February 2019, 40 patients were randomized: 19 to TOCC group and 21 to usual care group. The baseline characteristics for all patients are shown in Table 2. In the TOCC group, the intervention was completed in 84.2% (95% CI: 60.4–96.6%) and was not significantly different from the assumed completion rate of 75% (*p* = 0.438). The median length of time to complete TOCC intervention was 105 min (IQR: 75, 128). The three patients who did not have complete intervention by the nurse navigator in the TOCC group did not have a follow-up appointment scheduled because the family could not be contacted after discharge. There were no clinically significant differences in the baseline characteristics between those who completed the intervention and those who did not.

### Length of Stay

There was no significant difference in LOS between the TOCC group and the usual care group. Median LOS in the usual care group was 4.01 days (2.00, 10.45), while median LOS in the TOCC group was 5.95 days (4.02, 9.57). The original *p*-value was 0.138, and the FDR-adjusted *p*-value was 0.138.

### Patient Satisfaction

There was a trend toward higher patient satisfaction in the TOCC group as compared to the usual care group, but it did not reach statistical significance after adjustment for multiple comparisons. The median of total satisfaction score for the primary care group was 30 (26.00, 35.00), and the total satisfaction score for the TOCC group was 35.00 (33.00, 35.00). The original *p*-value was 0.050, and the FDR-adjusted *p*-value was 0.1.

### Associations Between Clinical Variables and LOS

**Figure 1:**
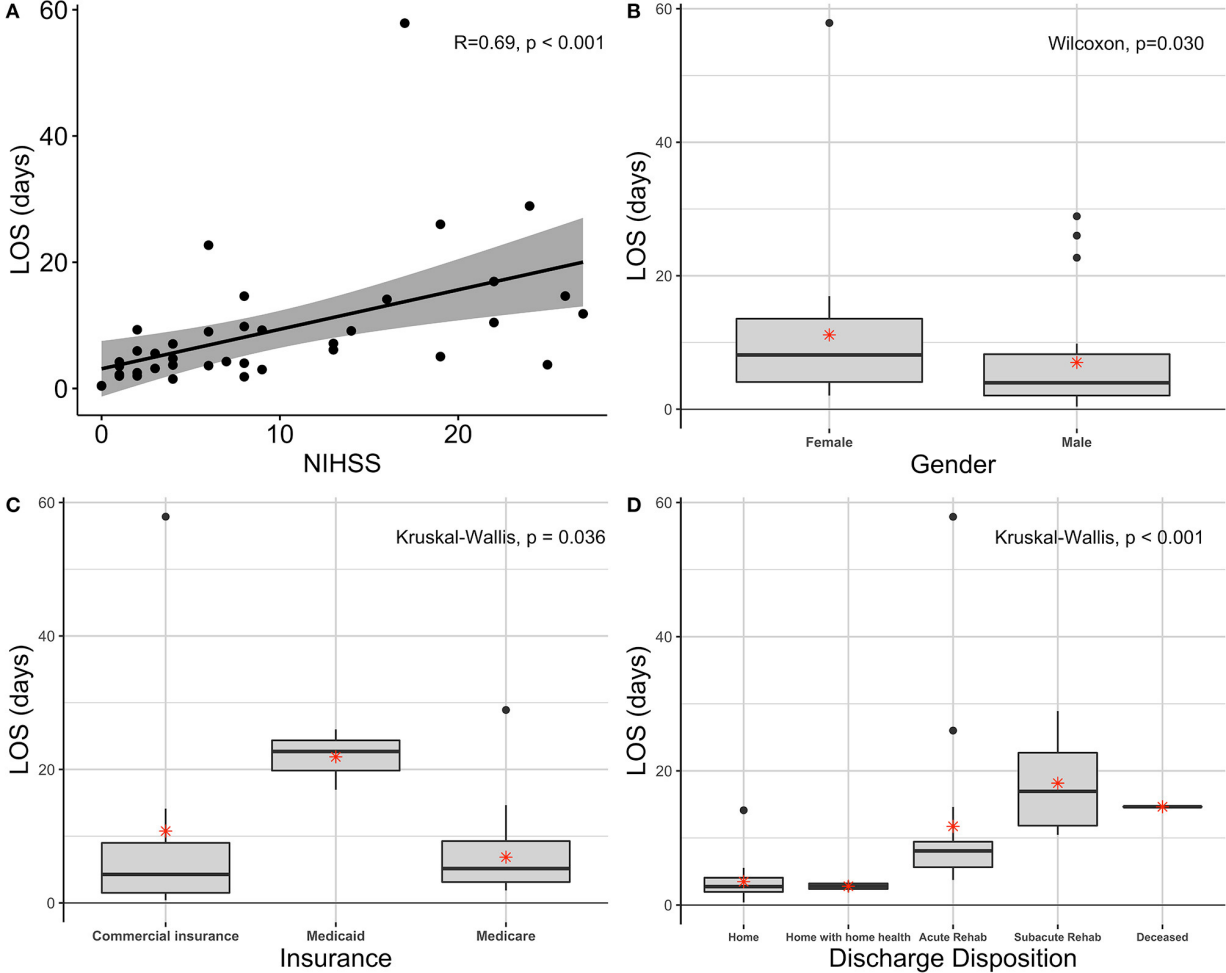
Associations between clinical variables and LOS. **(A)** Scatterplot of LOS and admission NIHSS, *r* = 0.69, *p* < 0.001. **(B)** Boxplot of LOS based on male and female sex, *p* = 0.030. **(C)** Boxplot of LOS compared to type of patient insurance (commercial vs. Medicaid *vs*. Medicare), *p* = 0.036. **(D)** Boxplot of LOS compared to final discharge disposition, *p* < 0.001. Boxplots include the median and interquartile range. The asterisk denotes the mean. LOS, length of stay; NIHSS, National Institutes of Health Stroke Scale.

The NIHSS, a measure of stroke severity, was significantly associated with LOS (*p*-value < 0.0001), with a higher NIHSS associated with a longer LOS (Figure 1A). The discharge mRS value was significantly associated with LOS (*p*-value 0.002), with a higher discharge mRS associated with a longer length of stay.

Gender and insurance status were both found to be significantly associated with LOS (*p*-value of 0.030 and 0.036, respectively). Specifically, female gender was associated with longer length of stay than male gender (Figure 1B). The median length of stay for female gender was 8.13 days (4.07, 13.55), while the median length of stay for male gender was found to be 3.96 days (2.05, 8.24). Medicaid insurance status was associated with longer LOS than Medicare or commercial insurance as demonstrated in Figure 1C. The median LOS for Medicaid was 22.70 days (19.82, 24.36), while the median length of stay for Medicare was 5.15 days (3.14, 9.27) and commercial insurance was 4.27 days (1.51, 9). The number of days between admission and date of PT/OT evaluations was significantly associated with LOS (*p*-value 0.001). The number of days between patient admission and SLP evaluation was not associated with LOS (*p*-value 0.108). The number of days between medically ready for discharge and date of rehabilitation referral placed was not significantly associated with LOS (*p*-value 0.202).

Final discharge disposition was significantly associated with LOS (*p*-value < 0.001), (Figure 1D). Specifically, discharge to subacute rehabilitation was associated with increased LOS compared to all other discharge dispositions. The median length of stay for discharge to subacute rehabilitation was 16.95 days (11.83, 22.7), whereas the median length of stay for discharge to home was 2.75 days (1.97, 4.07), discharge to home with home health was 2.80 days (2.39, 3,22), discharge to acute rehabilitation was 8.07 days (5.65, 9.44), and deceased was 14.65 days (only one patient).

Age, race, CCI, baseline mRS score, and home distance from hospital were not significantly associated with LOS (*p*-value 0.101, 0.596, 0.895, 0.9, and 0.167, respectively).

## Discussion

To our knowledge, this is the first prospective, randomized pilot study investigating a nurse-led TOCC program in hospitalized AIS patients. We demonstrated that it is feasible to implement a TOCC program with all portions of the intervention completed in 84.2% of the study population. The intervention did take an average of 1 h and 45 min to complete per patient, so in a large volume center, dedicated time quickly accumulates. However, our model was structured around a stroke-trained nurse who was able to provide a framework to ensure rapid stroke workup, timeliness of therapy evaluations, and supportive patient and family education. Previous literature, including the Transition Coaching for Stroke and Translational Stroke Clinic programs, utilized advanced practice providers to improve stroke prevention and compliance in the outpatient setting (15–17). By comparison, this study and others sought to improve transitions of care from the acute care hospital setting to other facilities and home, which is in line with the National Quality Forum and Institute of Medicine priorities (18–20). This TOCC study attempts to facilitate care throughout the duration of the hospitalization of the AIS patient.

There is some recent evidence that early supported discharge home with intensive rehabilitation services may be associated with shorter length of stay in ischemic stroke survivors (21). Although the TOCC intervention itself was not associated with a shorter LOS, we were able to identify patients at a higher risk for prolonged hospitalization. The factors associated with longer LOS were female sex, higher initial NIHSS, Medicaid insurance, and final disposition location. Of note is that the median NIHSS for female gender was higher than the median NIHSS for male gender (13 vs. 3.5), which may explain the difference in LOS by gender. Additionally, the median NIHSS for Medicaid patients was higher than that of Medicare and Commercial Insurance patients (19 vs. 7 and 6, respectively), which may explain in part why the median LOS for Medicaid was longer than that of Medicare and commercial insurance. An additional variable that contributed to LOS was the number of days between admission and date of PT/OT evaluations. Patients with more severe strokes and therefore higher NIHSS are likely less able to participate in PT/OT evaluations in the initial hospital days.

### Limitations

The limitations to this study include the fact that the research was conducted at a single center with a relatively small sample size, which may limit the generalizability of the results. The setting was a single academic, tertiary referral center with a large catchment area. The study's primary aim was to assess the feasibility of implementing a TOCC intervention, and it was not powered to evaluate statistically significant differences in LOS or patient satisfaction. Based on the data from this feasibility study, the mean LOS of the usual care group was 6.91 and 11.03 for the TOCC group, with a SD of 10.42, and would need 202 patients to achieve 80% power with α of 0.05. At our center, the usual care for AIS patients is in line with CSC standards, with each patient receiving coordinated care with a multidisciplinary team that includes a stroke nurse navigator. The high level of care provided to the usual care group may make detecting a difference in the two groups difficult. Based on the independent review of the study task completion, there was no definitive uptake of the TOCC intervention into the control group. However, given that the stroke nurse navigator was not blinded to the participants' group assignment, it is possible that a usual care group participant crossed over and inadvertently received elements of coordinated care intended for TOCC group. For future studies, we will plan to have separate stroke nurse navigators implementing the interventions for the study and control groups.

### Conclusion

We demonstrated that a transitions of care coordination program for hospitalized AIS patients is feasible and may be associated with higher patient satisfaction. There was no difference in hospital length of stay or patient satisfaction between the usual care group and our TOCC group. Hospitals designated as comprehensive stroke centers are often staffed with stroke nurse navigators, which allow for an easy implementation of nurse-driven quality improvement studies. With early identification of stroke patients at a higher risk for prolonged hospitalizations, such as those with severe strokes, with Medicaid insurance, and those referred to subacute rehabilitation, it is possible to better direct resources to these patients and families. A multi-site clinical trial with a large sample size to test the generalizability of this nurse-led TOCC model is planned.

## Data Availability Statement

The raw data supporting the conclusions of this article will be made available by the authors, without undue reservation.

## Ethics Statement

The studies involving human participants were reviewed and approved by Georgetown University IRB. The patients/participants provided their written informed consent to participate in this study.

## Author Contributions

MD: primary investigator, creating of protocol, construction of manuscript, data analysis, and editing. WZ: provided input of construction of manuscript, data analysis, and editing. RG and SP: participated in study protocol creation, construction of manuscript, data analysis, and editing. KM, AL, and JL: acted as nurse navigator in intervention group, participated in construction of manuscript, and editing. XG: provided statistical analysis of data obtained during study, participated in construction of manuscript, and editing. All authors contributed to the article and approved the submitted version.

## Conflict of Interest

The authors declare that the research was conducted in the absence of any commercial or financial relationships that could be construed as a potential conflict of interest.
